# Analysis of the effects of spaceflight and local administration of thrombopoietin to a femoral defect injury on distal skeletal sites

**DOI:** 10.1038/s41526-021-00140-0

**Published:** 2021-03-26

**Authors:** Ariane Zamarioli, Zachery R. Campbell, Kevin A. Maupin, Paul J. Childress, Joao P. B. Ximenez, Gremah Adam, Nabarun Chakraborty, Aarti Gautam, Rasha Hammamieh, Melissa A. Kacena

**Affiliations:** 1 Department of Orthopaedic Surgery, Indiana University School of Medicine, Indianapolis, IN USA; 2 Department of Orthopaedics and Anaesthesiology, Ribeirão Preto Medical School, Ribeirão Preto, SP Brazil; 3 Marian University College of Osteopathic Medicine, Indianapolis, IN USA; 4 Laboratory of Molecular Biology, Blood Center of Ribeirão Preto, Medical School, Ribeirão Pre, SP Brazil; 5 Medical Readiness Systems Biology, Walter Reed Army Institute of Research, Silver Spring, MD USA; 6 Geneva Foundation, Walter Reed Army Institute of Research, Silver Spring, MD USA; 7 Richard L. Roudebush VA Medical Center, Indianapolis, IN USA

**Keywords:** Translational research, Physiology

## Abstract

With increased human presence in space, bone loss and fractures will occur. Thrombopoietin (TPO) is a recently patented bone healing agent. Here, we investigated the systemic effects of TPO on mice subjected to spaceflight and sustaining a bone fracture. Forty, 9-week-old, male, C57BL/6 J were divided into 4 groups: (1) Saline+Earth; (2) TPO + Earth; (3) Saline+Flight; and (4) TPO + Flight (*n* = 10/group). Saline- and TPO-treated mice underwent a femoral defect surgery, and 20 mice were housed in space (“Flight”) and 20 mice on Earth for approximately 4 weeks. With the exception of the calvarium and incisor, positive changes were observed in TPO-treated, spaceflight bones, suggesting TPO may improve osteogenesis in the absence of mechanical loading. Thus, TPO, may serve as a new bone healing agent, and may also improve some skeletal properties of astronauts, which might be extrapolated for patients on Earth with restraint mobilization and/or are incapable of bearing weight on their bones.

## Introduction

The emergence of commercialized space companies, NASA’s Artemis Program and Space Launch System, and continued development of additional national space programs are driving human spaceflight to greater horizons^1–5^. Missions planned to planetary bodies such as the Moon and Mars are in early stages with the intent of launching in the coming decades. These missions seek to establish more permanent human habitation on orbital stations and potential planetary colonies, which will require a surge of human participation^6^. The human component of spaceflight, particularly the physiological changes induced by the absence of the Earth’s gravity, remains key in establishing the feasibility of long-duration space missions. The year-long study of twin astronauts Mark and Scott Kelly demonstrated long-duration spaceflight has effects ranging from ocular disturbances, significant radiation exposure resulting in epigenetic changes, and changes in muscle and bone^7^.

It is well documented that a consequence of microgravity is the reduction of weight loading on the skeletal system which leads to reduced bone mineral density (BMD). Variation on BMD loss is high among individuals and individual bones. A study of Cosmonauts on the International Space Station (ISS) showcased this variability. Seven out of eight experienced a reduction in BMD (2.5–10.6%) in the lumbar vertebrae, all eight showed decreased BMD in the femur (3–10%), and four of eight showed a 1.7–10.5% decrease in BMD in the femoral neck^8^. Another study demonstrated that exposure to the microgravity environment of space resulted in losses in the spine, femoral neck, trochanter, and pelvis of about 1–1.6%, with considerable variation between individuals^9^. Recent research has corroborated the negative effects of spaceflight on BMD^10–13^. As BMD is lost, structural integrity is compromised leading to increased potential of fracture.

Fracture risk is thought to be minimal in low Earth orbit because of the virtual nonexistence of applied load injuries to astronauts such as crushing or falling events^14^. Other potential risks are mitigated by engineering and design centered around preserving human safety. Fracture risk becomes more prevalent when factoring in activities that increase the mechanical load on bones. Mission critical activities such as construction of habitats, repairs, extra-vehicular activities, and performance in bulkier extra-vehicular activity suits add extra risk. Furthermore, future missions that require exploratory components on the surface of other planetary bodies after long-duration zero-gravity will add a mechanical load due to subsequent re-entry into a gravity environment (or partial gravity such as that of the Moon or Mars) which in turn increases fracture risk. Indeed, predictive models of fracture risk for astronauts indicate risk of fractures to the hip and wrist are high^15,16^. Therefore, understanding the systemic response to fractures and their management in a microgravity environment is of critical importance in the preparation of future, longer duration, spacefaring missions, and habitation. Preventative measures that reduce BMD loss such as dietary supplements, exercise with specialized equipment such as the advanced resistance exercise device (ARED) on the ISS, pharmacological treatment, and reducing risky behavior correlated with potential fractures is standard^17–21^.

With longer duration space habitation or colonization of planets, it is likely that fractures will occur and will require treatment in spaceflight. As a result, investigators have examined a variety of treatment options including administration of parathyroid hormone and low-intensity ultrasound^22,23^. However, parathyroid hormone injections remain controversial as studies have indicated that long duration or large dose-dependent treatment increases risk for developing osteosarcoma and hypercalcemia^24–26^. Furthermore, low intensity ultrasound has shown little benefit in radiographic bone healing, improvement in days to weight-loading, or pain experienced^27^. On the other hand, for larger fractures an internal fixation device is the primary option. Currently, administration of bone morphogenetic protein-2 (BMP-2) is commonly used to help with bone healing^28,29^. However, some studies have indicated that BMP-2, while a potent activator of bone growth, has multiple side effects such as seroma formation, bone overgrowth, osteolysis, and an increased risk of developing cancer^30,31^. Therefore, identifying alternative bone healing agents is of great importance for patients on Earth and in space alike. Our laboratory recently patented the use of the main megakaryocyte (MK) growth factor, thrombopoietin (TPO), as a novel bone healing agent, and has previously demonstrated TPO’s ability to promote bone regeneration in mice, rats, and pigs on Earth^32^.

This study is a component of the Rodent Research 4 (RR4) mission with the primary objectives being to better understand bone healing and bone tissue regeneration and to study the impacts of microgravity on these processes. Specifically, due to restrictions of housing capacity on the ISS and the difficulties posed by conducting research in spaceflight, specimens are limited and are valuable resources. For this reason, it is important to extract as much data as possible from each specimen for analysis. Indeed, previously our laboratory demonstrated that distal skeletal sites experienced systemic effects from femoral fracture surgery in spaceflight^33^. Here we are examining the effects of gravity as well as the systemic effects of local TPO administration at the femoral fracture site and on several distal bones. Specifically, we examined bones from the limbs (humerus and tibia), torso (L4 vertebrae, 10th rib, and 3rd body of the sternum), and skull (calvaria, mandible, and incisor). We hypothesized that a single application of TPO at the fracture gap would lead to systemic effects at distal bone sites in mice, and that these effects would be different in mice flown in space versus those housed on the Earth.

## Results

### Experimental overview

We classified our outcomes into two groups according to their weight-bearing status: (1) non-weight-bearing bones—calvarium, mandible, incisor, rib, and sternum and; (2) weight-bearing bones—vertebra, humerus, and tibia. We used two-way ANOVA analyses in order to identify which variable (TPO or microgravity) exerted a significant main effect on our comparisons. Furthermore, for each bone, we report significant (*p* < 0.05) and trending data (*p* < 0.09) for comparisons between groups in the same Habitat (i.e. Saline + Earth versus TPO + Earth or Saline + Flight versus TPO + Flight) to understand the effects of treatment both on Earth and in flight as well as comparisons between spaceflight and Earth to assess the influence of gravity on the treatment (i.e. TPO + Earth versus TPO + Flight or Saline + Earth versus Saline + Flight). For ease of readability, in the sections below, for each bone examined, we present the results in the following order. First, we present data comparing TPO to saline treatment on Earth. Second, we compare outcomes from mice treated with TPO to those treated with saline in space. Third, we examine the impact of microgravity on saline-treated mice by comparing specimens from Earth and Flight groups. Fourth, we examine the impact of spaceflight on TPO treatment by comparing bones from Earth and Flight groups. Finally, we describe whether the significant main effect was the microgravity, TPO or both, alongside the interaction between them.

### Non-weight bearing bones (Table 1)

#### Calvarium

With respect to the calvarium, TPO treatment of femurs at the time of surgery, did not show any significant difference when administered on Earth when compared with the saline group. In fact, it even worsened some parameters (i.e. 48% larger marrow volume and 44% higher trabecular separation). Similarly, TPO treatment in flight also showed a 2% lower fractional bone volume (BV/TV, *p* = 0.02), a 14% decrease in cortical thickness (Ct.Th, *p* = 0.05) and a 12% reduction in trabecular thickness (Tb.Th) than observed with saline treatment (*p* = 0.03), associated with a two-fold increase in marrow volume (MV) and trabecular separation (Tb.Sp, *p* = 0.02 and *p* = 0.03, respectively). One intriguing outcome refers to a 12% increase in trabecular number (Tb.N, *p* = 0.04) due to TPO administration, which may be related to trabecular bone resorption into smaller fragments. In the comparison between ground and spaceflight, the exposure to microgravity within the saline group resulted in higher BV/TV (+5%, *p* = 0.01), Ct.Th (+22%, *p* = 0.02) and Tb.Th (+22%, *p* = 0.008), associated with lower MV (−99%, *p* = 0.02), lower Tb.Sp (−85%, *p* = 0.01), and a reduction in Tb.N (−13%, *p* = 0.02) than on Earth. In the comparison between Earth and flight within the TPO group (TPO + Earth versus TPO + Flight), the exposure to microgravity trended towards an increase in BV/TV (+4%, *p* = 0.07) and lower trabecular separation (−54%, *p* = 0.08) than on the ground. Accordingly, our two-way ANOVA analyses showed a significant main effect of microgravity in the BV/TV, MV, Ct.Th, Tb.Th, Tb.Sp, and Tb.N at the calvarium (*p* < 0.003).

**Table 1:** Bone microstructural morphometry assessed by μCT in non-weight-bearing skeletal sites.

	Earth		Flight	
	Saline	TPO	Saline	TPO
*Calvarium*				
TV (mm^3^)	0.058 ± 0.003	0.058 ± 0.006	0.053 ± 0.005	0.053 ± 0.004
BV (mm^3^)	0.054 ± 0.003	0.054 ± 0.003	0.053 ± 0.005	0.051 ± 0.004
BV/TV (%)	94.496 ± 2.709^#^	93.831 ± 3.501	99.370 ± 0.116^b^	97.406 ± 1.267^a,b^^’^
MV (mm^3^)	0.003 ± 0.002^#^	0.004 ± 0.002	0.0003 ± 0.00007^b^	0.001 ± 0.001^a^
Width (mm)	0.166 ± 0.008	0.166 ± 0.017	0.155 ± 0.012	0.152 ± 0.011
Ct.Th (mm)	0.096 ± 0.008^#^	0.093 ± 0.010	0.117 ± 0.100^b^	0.101 ± 0.002^a^
Tb.Th (mm)	0.075 ± 0.006^#^	0.073 ± 0.008	0.090 ± 0.006^b^	0.079 ± 0.002^a^
Tb.Sp (mm)	0.004 ± 0.002^#^	0.005 ± 0.002	0.001 ± 0.0001^b^	0.002 ± 0.001^a,b’^
Tb.N (1/mm)	12.690 ± 0.618^#^	12.979 ± 0.910	11.024 ± 0.836^b^	12.359 ± 0.296^a^
*Mandible*				
T.Ar (mm^2^)	1.943 ± 0.020	1.923 ± 0.061	1.946 ± 0.045	1.914 ± 0.059
B.Ar (mm^2^)	1.311 ± 0.021	1.331 ± 0.055	1.337 ± 0.041	1.340 ± 0.051
M.Ar (mm^2^)	0.632 ± 0.041*	0.592 ± 0.037	0.609 ± 0.029	0.574 ± 0.011^a^
B.Ar/T.Ar (%)	67.498 ± 1.781	69.231 ± 1.715	68.719 ± ± 1.336	70.008 ± 0.578
CEJ-ABC (mm)	0.192 ± 0.008	0.213 ± 0.025	0.221 ± 0.027	0.221 ± 0.025
*Incisor*				
T.Ar (mm^2^)	0.467 ± 0.005	0.484 ± 0.022	0.481 ± 0.015	0.471 ± 0.010
E + D.Ar (mm^2^)	0.377 ± 0.034	0.421 ± 0.039	0.394 ± 0.037	0.380 ± 0.032
Pu.Ar (mm^2^)	0.090 ± 0.029	0.063 ± 0.018	0.087 ± 0.023	0.091 ± 0.028
E + D.Ar/T.Ar (%)	80.709 ± 6.539	86.857 ± 4.122	81.742 ± 5.355	80.593 ± 6.159
*Rib*				
T.Ar (mm^2^)	0.088 ± 0.024^Ψ^	0.103 ± 0.012	0.114 ± 0.013^b’^	0.089 ± 0.014^a^
B.Ar (mm^2^)	0.069 ± 0.017	0.075 ± 0.007	0.083 ± 0.008	0.069 ± 0.007^a^
M.Ar (mm^2^)	0.019 ± 0.008^Ψ^	0.028 ± 0.005	0.031 ± 0.005^b’^	0.020 ± 0.007^a^
B.Ar/T.Ar (%)	78.766 ± 3.455^Ψ^	72.787 ± 1.884^a^	72.875 ± 2.140^b^	77.601 ± 4.068^a’,b’^
Ct.Th (mm)	0.081 ± 0.006	0.079 ± 0.002	0.081 ± 0.002	0.081 ± 0.004
*Sternum*				
TV (mm^3^)	0.527 ± 0.029*^’^	0.512 ± 0.036	0.560 ± 0.045	0.518 ± 0.026
BV (mm^3^)	0.058 ± 0.012	0.045 ± 0.012	0.051 ± 0.006	0.050 ± 0.006
BV/TV (%)	10.994 ± 1.647^Ψ^	8.796 ± 1.684^a’^	9.047 ± 0.751^b^	9.724 ± 1.038
BS/BV (%)	0.084 ± 0.004	0.086 ± 0.006	0.088 ± 0.002^b’^	0.086 ± 0.006
Tb.Th (mm)	0.039 ± 0.002	0.040 ± 0.004	0.038 ± 0.001	0.040 ± 0.001^a^
Tb.Sp (mm)	0.211 ± 0.017	0.243 ± 0.024^a^	0.214 ± 0.022	0.212 ± 0.024 ^a,b’^
Tb.N (1/mm)	2.770 ± 0.294*^’,Ψ^	2.198 ± 0.337^a^	2.380 ± 0.239^b^	2.436 ± 0.273
SMI	1.718 ± 0.168*	1.908 ± 0.140	1.774 ± 0.119	1.964 ± 0.301
Conn.D (1/μm^3^)	0.028 ± 0.008	0.020 ± 0.007	0.026 ± 0.005	0.028 ± 0.013
Ct.Th (mm)	0.081 ± 0.006	0.079 ± 0.002	0.081 ± 0.002	0.081 ± 0.004

Values are expressed as mean ± SD. TV tissue volume, BV bone volume, BV/TV bone volume fraction, MV marrow volume, calculated as TV−BV, Ct.Th average cortical thickness, Tb.Th trabecular thickness, Tb.Sp trabecular separation, Tb.N trabecular number, T.Ar tissue area, B.Ar bone area, M.Ar marrow area, calculated as T.Ar−B.Ar, B.Ar/T.Ar bone area fraction, CEJ-ABC lingual cementum–enamel to alveolar bone crest distance, E+D.Ar enamel + dentin area, Pu.Ar pulp area, E+D.Ar/T.Ar enamel + dentin area fraction, BS/BV specific bone surface, SMI structure model index, Conn.D connectivity density. Sample sizes for all bones are *n* = 5 unless otherwise indicated: Calvarium (TPO + Earth and Saline + Flight: *n* = 4), Mandible and Incisor (TPO + Earth: *n* = 4, TPO + Flight: *n* = 3), Rib (TPO + Earth: *n* = 3, TPO + Flight: *n* = 4). Significant interactions were detected by two-way ANOVA followed by Tukey post-hoc analyses and were designated by * when a significant main effect of TPO was detected (or a trend towards significance designated by *’ when *p* < 0.09); by ^#^ when a significant main effect of microgravity was detected; or by ^Ψ^ when a significant TPO × microgravity interaction was detected. Significant differences were also based on: (1) TPO treatment (e.g., TPO + Earth versus Saline + Earth or TPO + Flight versus Saline + Flight, designated by ^a^ when *p* < 0.05 indicating a significant difference or ^a’^ when *p* < 0.09 indicating a trend towards significant difference); or (2) microgravity exposure with or without treatment (e.g., Saline + Earth versus Saline + Flight or TPO + Earth versus TPO + Fight, designated by ^b^ when *p* < 0.05 indicating a significant difference or ^b’^ when *p* < 0.09 indicating a trend towards significant difference).

#### Mandible

TPO administration to the femur at the time of surgery, did not impact the mice housed on Earth. However, the flight, TPO-treated group showed a decrease in M.Ar compared to that observed with saline treatment (−6%, *p* = 0.04). Gravity changes did not significantly impact bone parameters for saline-treated or TPO-treated mandibles. Corroborating these findings, our two-way ANOVA analyses showed a significant main effect of TPO in the M.Ar at the mandible (*p* = 0.03).

#### Incisor

With regard to the incisor, we did not detect any significant changes in bone parameters due to TPO treatment or gravity conditions.

#### Rib

Mice treated with TPO at the time of femoral fracture surgery exhibited an 8% lower bone area/tissue area (B.Ar/T.Ar) in the ribs than did saline-treated mice on Earth (*p* = 0.02). Out of the several bones examined, the rib was the only one that exhibited some conflicting bone parameter data regarding the group submitted to spaceflight and TPO treatment. Specifically, in mice treated with TPO and housed in space, there was a discrete reduction in total and bone area compared to that observed in saline-treated mice housed in space (*p* = 0.03). However, since this reduction was higher in T.Ar than in B.Ar, there was a significant 35% reduction in M.Ar (*p* = 0.03) in TPO-treated mice when compared to saline treatment, which led to a trend towards a higher B.Ar/T.Ar (+7%, *p* = 0.06). In the comparison between ground and spaceflight, the exposure to microgravity within the saline group resulted in a lower B.Ar/T.Ar (−8%, *p* = 0.02) than on Earth and trended towards higher T.Ar (+77%, *p* = 0.08) and M.Ar (+61%, *p* = 0.09). Within the TPO group, a trend towards a higher B.Ar/T.Ar (+7%, *p* = 0.06) was seen in the mice exposed to microgravity in comparison to those on Earth. Accordingly, our two-way ANOVA analyses showed a significant TPO × microgravity interaction in T.Ar, M.Ar, and B.Ar/T.Ar in the rib (*p* < 0.04).

#### Sternum

Similarly to the calvarium and the rib, TPO treatment, at the time of femoral fracture surgery, seems to differentially alter bone parameters in spaceflown mice compared to those housed on Earth. Specifically, on Earth, the TPO group exhibited a 15% increase in Tb.Sp (*p* = 0.04), associated with a 22% reduction in Tb.N (*p* = 0.02), and a trend towards a lower BV/TV (−20%, *p* = 0.07) than seen in the saline group. Conversely, in spaceflight, TPO increased the Tb.Th and decreased Tb.Sp in comparison with the saline group (*p* = 0.04 and *p* = 0.05, respectively). Within the saline group, significant decreases in BV/TV and Tb.N (−18%, *p* = 0.04 and −14%, *p* = 0.05), associated with trends toward a higher specific bone surface (BS/BV, +5%, *p* = 0.09), were found in the spaceflight group when compared to the Earth group. Within the TPO group, the microgravity samples trended towards lower trabecular separation when compared to the samples on Earth (−19%, *p* = 0.07). Our two-way ANOVA analyses showed a significant main effect of TPO on SMI (*p* = 0.04) and a trend on TV (*p* = 0.08) and Tb.N (*p* = 0.06) in the sternum. Furthermore, a significant TPO × microgravity interaction was detected in BV/TV and Tb.N (*p* < 0.03).

### Weight-bearing bones (Tables 2 and 3)

#### Vertebra (Table 2, Fig. 1)

Next, we examined the effects of spaceflight and TPO administration at the femoral fracture site on the L4 vertebra. On Earth, although the TPO group exhibited lower connectivity density (Conn.D) than saline (−71%, *p* = 0.02), no difference was observed in the remaining parameters. In flight, TPO increased Tb.N by 11% (*p* = 0.04) when compared to saline samples. In the comparison between ground and spaceflight, the exposure to microgravity within the saline group led to a decrease in Conn.D when compared to the ground samples (−72%, *p* = 0.02). No significant differences in bone parameters were detected when comparing TPO-treated microgravity and Earth specimens. Accordingly, our two-way ANOVA analyses showed a significant main effect of TPO on Tb.N (*p* = 0.05), and Conn.D (*p* = 0.03) at the L4 vertebra, associated with a trend toward significance for Tb.Sp (*p* = 0.07). Furthermore, the parameter Conn.D also showed a trend towards a significant main effect of microgravity (*p* = 0.07) and a significant TPO × microgravity interaction (*p* = 0.008).

**Table 2:** Bone microstructural morphometry assessed by μCT in partial weight-bearing skeletal sites.

	Earth		Flight	
	Saline	TPO	Saline	TPO
*L4*				
TV (mm^3^)	0.950 ± 0.245	1.062 ± 0.053	1.110 ± 0.086	1.042 ± 0.062
BV (mm^3^)	0.181 ± 0.066	0.202 ± 0.029	0.176 ± 0.024	0.184 ± 0.023
BV/TV (%)	18.516 ± 4.072	19.031 ± 2.878	15.865 ± 1.510	17.637 ± 1.566
BS/BV (%)	70.457 ± 10.017	72.244 ± 8.310	78.063 ± 4.077	79.384 ± 13.137
Tb.Th (mm)	0.048 ± 0.006	0.048 ± 0.006	0.045 ± 0.004	0.044 ± 0.003
Tb.Sp (mm)	0.200 ± 0.006*^’^	0.193 ± 0.008	0.210 ± 0.016	0.184 ± 0.029
Tb.N (1/mm)	3.777 ± 0.395*	3.963 ± 0.195	3.501 ± 0.176	3.983 ± 0.406^a^
SMI	1.274 ± 0.114	1.170 ± 0.062	1.222 ± 0.105	1.401 ± 0.441
Conn.D (1/µm^3^)	0.178 ± 0.079*^,#’,Ψ^	0.052 ± 0.004^a^	0.050 ± 0.008^b^	0.074 ± 0.065
*Trabecular humerus*				
TV (mm^3^)	0.659 ± 0.052	0.709 ± 0.031^a^	0.661 ± 0.052	0.655 ± 0.031^b^
BV (mm^3^)	0.083 ± 0.033	0.112 ± 0.037^a’^	0.074 ± 0.029	0.088 ± 0.019
BV/TV (%)	12.603 ± 5.249	15.658 ± 4.763	11.312 ± 4.635	13.484 ± 2.806
BS/BV (%)	68.294 ± 9.754	62.610 ± 4.915	66.825 ± 10.167	65.999 ± 4.618
Tb.Th (mm)	0.062 ± 0.006	0.065 ± 0.004	0.063 ± 0.007	0.064 ± 0.003
Tb.Sp (mm)	0.240 ± 0.043*	0.217 ± 0.026	0.274 ± 0.059	0.212 ± 0.023^a^
Tb.N (1/mm)	1.962 ± 0.705	2.364 ± 0.583	1.739 ± 0.589	2.100 ± 0.383
SMI	2.585 ± 0.218	2.434 ± 0.184	2.586 ± 0.142	2.594 ± 0.100^b^
Conn.D (1/μm^3^)	0.282 ± 0.139*^,#^	0.394 ± 0.168	0.181 ± 0.087	0.283 ± 0.095^a^
*Cortical humerus*				
T.Ar (mm^2^)	0.913 ± 0.048	0.900 ± 0.043	0.946 ± 0.028	0.912 ± 0.047
B.Ar (mm^2^)	0.548 ± 0.041	0.530 ± 0.046	0.544 ± 0.030	0.537 ± 0.040
M.Ar (mm^2^)	0.365 ± 0.028^#,Ψ’^	0.371 ± 0.017	0.403 ± 0.033^b^	0.375 ± 0.012^a^
B.Ar/T.Ar (%)	60.015 ± 2.632	58.633 ± 2.726	57.459 ± 2.967^b’^	58.797 ± 1.652
Ct.Th (mm)	0.181 ± 0.013	0.174 ± 0.014	0.176 ± 0.012	0.177 ± 0.011

Values are expressed as mean ± SD. TV: tissue volume, BV: bone volume; BV/TV: bone volume fraction; BS/BV: specific bone surface; Tb.Th: trabecular thickness; Tb.Sp: trabecular separation; Tb.N: trabecular number; SMI: structure model index; Conn.D: connectivity density; T.Ar: tissue area; B.Ar: bone area; M.Ar: marrow area; B.Ar/T.Ar: bone area fraction; Ct.Th: average cortical thickness. Sample sizes for L4 are *n* = 5 for all groups, with the exception of Saline+Flight, *n* = 4. For humerus sample sizes are *n* = 10, with the exception of TPO + Earth, *n* = 5. Significant interactions were detected by two-way ANOVA followed by Tukey post-hoc analyses and were designated by * when a significant main effect of TPO was detected (or a trend towards significance designated by *’ when *p* < 0.09); by ^#^ when a significant main effect of microgravity was detected (or a trend towards significance designated by ^#^’ when *p* < 0.09); or by ^Ψ^ when a significant TPO × microgravity interaction was detected (or a trend towards significance designated by ^Ψ^’ when *p* < 0.09). Significant differences were also based on: (1) TPO treatment (e.g., TPO + Earth versus Saline + Earth or TPO + Flight versus Saline + Flight, designated by ^a^ when *p* < 0.05 indicating a significant difference; or (2) microgravity exposure with or without treatment (e.g., Saline + Earth versus Saline + Flight or TPO + Earth versus TPO + Fight, designated by ^b^ when *p* < 0.05 indicating a significant difference or ^b’^ when *p* < 0.09 indicating a trend towards significant difference).

**Table 3:** Bone microstructural morphometry assessed by μCT in weight-bearing skeletal sites.

	Earth		Flight	
	Saline	TPO	Saline	TPO
*Trabecular tibia*				
TV (mm^3^)	1.339 ± 0.083^#’^	1.427 ± 0.094	1.461 ± 0.101	1.450 ± 0.068
BV (mm^3^)	0.241 ± 0.043	0.244 ± 0.033	0.277 ± 0.136^b’^	0.191 ± 0.030^b^
BV/TV (%)	18.068 ± 3.463	17.180 ± 2.582	18.918 ± 8.874	13.228 ± 2.175^b^
Tb.Th (mm)	0.047 ± 0.005*^’,Ψ’^	0.047 ± 0.004	0.055 ± 0.014	0.041 ± 0.002^b^
Tb.Sp (mm)	0.145 ± 0.012^#’^	0.153 ± 0.012	0.160 ± 0.013	0.156 ± 0.006
Tb.N (1/mm)	6.348 ± 0.464^#’^	6.121 ± 0.443	5.787 ± 0.291^b’^	6.074 ± 0.209
SMI	2.353 ± 0.301	2.295 ± 0.217	2.290 ± 0.509	2.529 ± 0.174
Conn.D (1/mm^3^)	204.232 ± 55.828	184.352 ± 56.805	141.417 ± 46.682	160.358 ± 39.656
*Cortical tibia*				
T.Ar (mm^2^)	0.989 ± 0.041^Ψ’^	1.025 ± 0.048	1.064 ± 0.070	1.029 ± 0.081
B.Ar (mm^2^)	0.676 ± 0.043^Ψ’^	0.710 ± 0.052	0.738 ± 0.053	0.695 ± 0.020
M.Ar (mm^2^)	0.313 ± 0.010	0.315 ± 0.026	0.326 ± 0.026	0.324 ± 0.011
B.Ar/T.Ar (%)	53.265 ± 3.475	55.264 ± 1.946	55.622 ± 3.731	52.64 ± 2.927
Ct.Th (mm)	0.246 ± 0.014	0.255 ± 0.022	0.258 ± 0.013	0.248 ± 0.005
Density TV (1/cm)	3.029 ± 0.209^Ψ’^	3.109 ± 0.098	3.154 ± 0.133	2.962 ± 0.127^b’^
Density BV (1/cm)	5.117 ± 0.118	5.110 ± 0.075	5.087 ± 0.046	5.105 ± 0.031

Values are expressed as mean ± SD. TV: tissue volume, BV: bone volume; BV/TV: bone volume fraction; BS/BV: specific bone surface; Tb.Th: trabecular thickness; Tb.Sp: trabecular separation; Tb.N: trabecular number; SMI: structure model index; Conn.D: connectivity density; T.Ar: tissue area; B.Ar: bone area; M.Ar: marrow area; B.Ar/T.Ar: bone area fraction; Ct.Th: average cortical thickness. Sample sizes for all bones are *n* = 5. Significant interactions were detected by two-way ANOVA followed by Tukey post-hoc analyses and were designated by *’ when a trend towards a significant main effect of TPO was detected (*p* < 0.09), by ^#’^ when a trend towards a significant main effect of microgravity was detected (*p* < 0.09), or by ^Ψ’^ when a trend towards a significant TPO × microgravity interaction was detected (*p* < 0.09). Significant differences were also based on microgravity exposure with or without treatment (e.g., Saline+Earth versus Saline+Flight or TPO + Earth versus TPO + Fight, designated by ^b^ when *p* < 0.05 indicating a significant difference or ^b’^ when *p* < 0.09 indicating a trend towards significant difference).

**Fig. 1:**
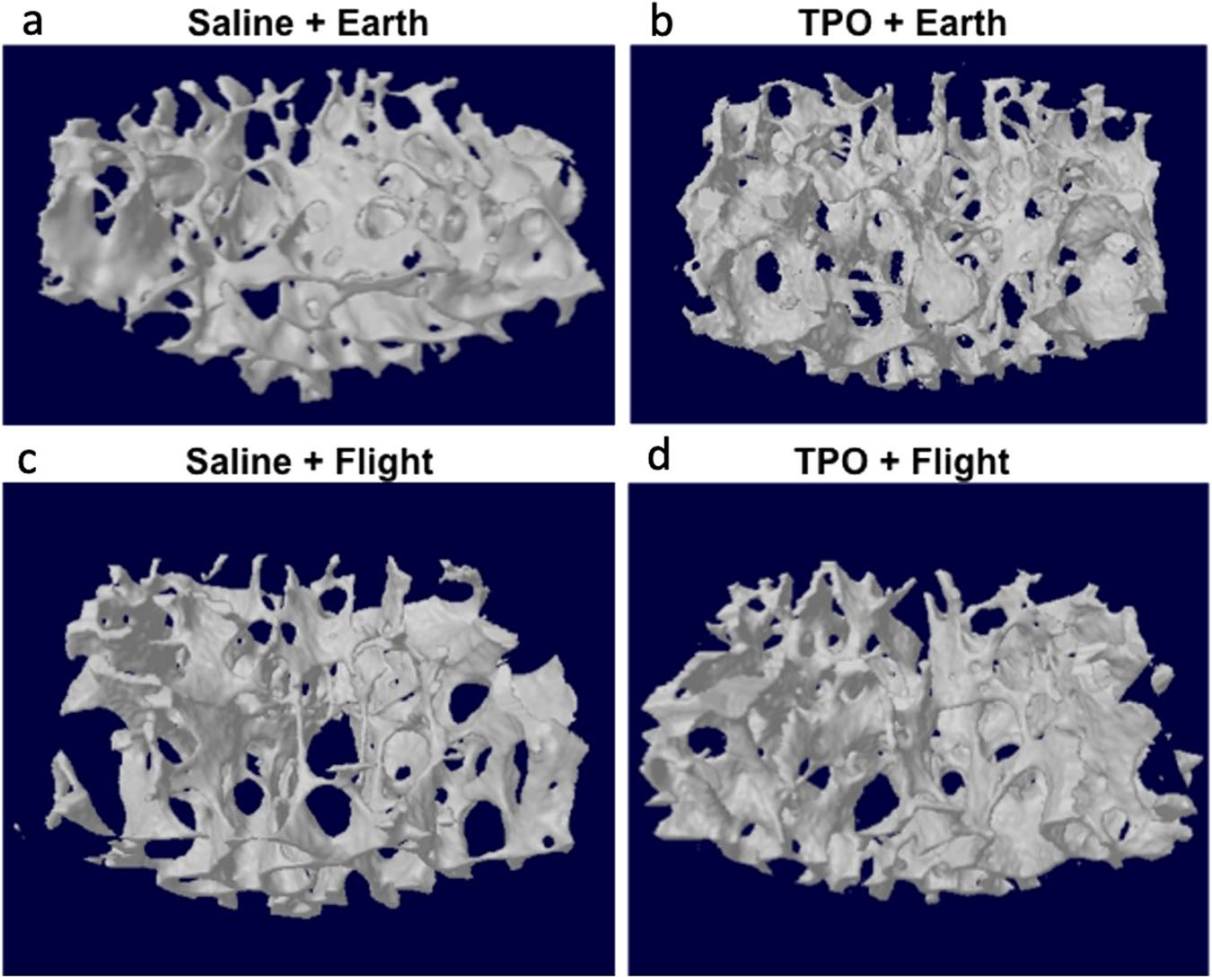
Representative 3D µCT reconstructions of the trabecular compartment of the L4 vertebra. **a** Saline + Earth; **b** TPO + Earth; **c** Saline + ; and **d** TPO + Flight. Tridimensional microstructural assessment of the vertebra evidencing increments in trabecular number in the TPO + Flight group as compared to the Saline+Flight group. TPO did not exhibit such osteogenic effects on Earth.

#### Trabecular humerus (Table 2)

We then examined the effects of spaceflight and femoral fracture TPO treatment on the humerus. With regard to trabecular humerus parameters, on Earth, the TPO group evidenced higher TV (+9%, *p* = 0.03) than saline treatment, trended towards a higher BV (+8%, *p* = 0.06), and a non-significant increase in Tb.N (+20%). In flight, TPO resulted in lower Tb.Sp and higher Conn.D than saline treatment (−22%, *p* = 0.01 and +50%, *p* = 0.03, respectively), and in a non-significant higher Tb.N (+21%). When examining Earth versus flight, no differences were observed in saline-treated mice. However, in the comparison between ground and spaceflight, the exposure of the TPO group to microgravity resulted in lower total volume (TV) and higher structure model index (SMI) than on Earth (−9%, *p* = 0.01 and +7%, *p* = 0.04, respectively). Furthermore, our two-way ANOVA analyses showed a significant main effect of TPO on Tb.Sp (*p* = 0.006) and Conn.D (*p* = 0.05), in which Conn.D also exhibited a significant main effect of microgravity (*p* = 0.02) within the trabecular compartment of the humerus.

#### Cortical humerus (Table 2)

With regard to the cortical bone component of the humerus, TPO treatment did not show any significant difference when administered on Earth when compared with the saline group. In flight, TPO decreased the M.Ar when compared to saline (−8%, *p* = 0.04). In the comparison between ground and spaceflight, the exposure to microgravity within the saline group led to a higher M.Ar and trended towards lower B.Ar/T.Ar than on the ground (+11%, *p* = 0.02 and −5%, *p* = 0.07). No differences were detected when comparing Earth and flight mice treated with TPO. Additionally, our two-way ANOVA analyses showed a significant main effect of microgravity on M.Ar (*p* = 0.01), alongside a trend towards a significant TPO × microgravity interaction (*p* = 0.06) within the cortical compartment of the humerus.

#### Trabecular tibia (Table 3, Fig. 2)

We next examined the impact of spaceflight and local TPO administration to the femur fracture on the tibia. With regard to the trabecular compartment of the tibia, TPO treatment did not result in significant differences when administered on Earth as compared with saline treatment. In flight, although TPO did not show significant differences, it showed a trend at increasing Tb.N and Conn.D (*p* > 0.09). In the comparison between ground and spaceflight, the exposure to microgravity within the saline group trended toward an increase in BV (+8%, *p* = 0.07) and a decrease in Tb.N (−9%, *p* = 0.05) than on ground. Within the TPO group, microgravity exposure resulted in lower BV (−21%, *p* = 0.03), BV/TV (−24%, *p* = 0.03), and Tb.Th (−15%, *p* = 0.01) than on Earth. Further, our two-way ANOVA analyses showed a trend towards a significant main effect of microgravity on TV (*p* = 0.08), Tb.Sp (*p* = 0.09), and Tb.N (*p* = 0.08) within the trabecular compartment of the tibia. A trend towards a significant main effect of TPO was also detected on Tb.Th (*p* = 0.06), which also trended towards a significant TPO × microgravity interaction (*p* = 0.06).

**Fig. 2:**
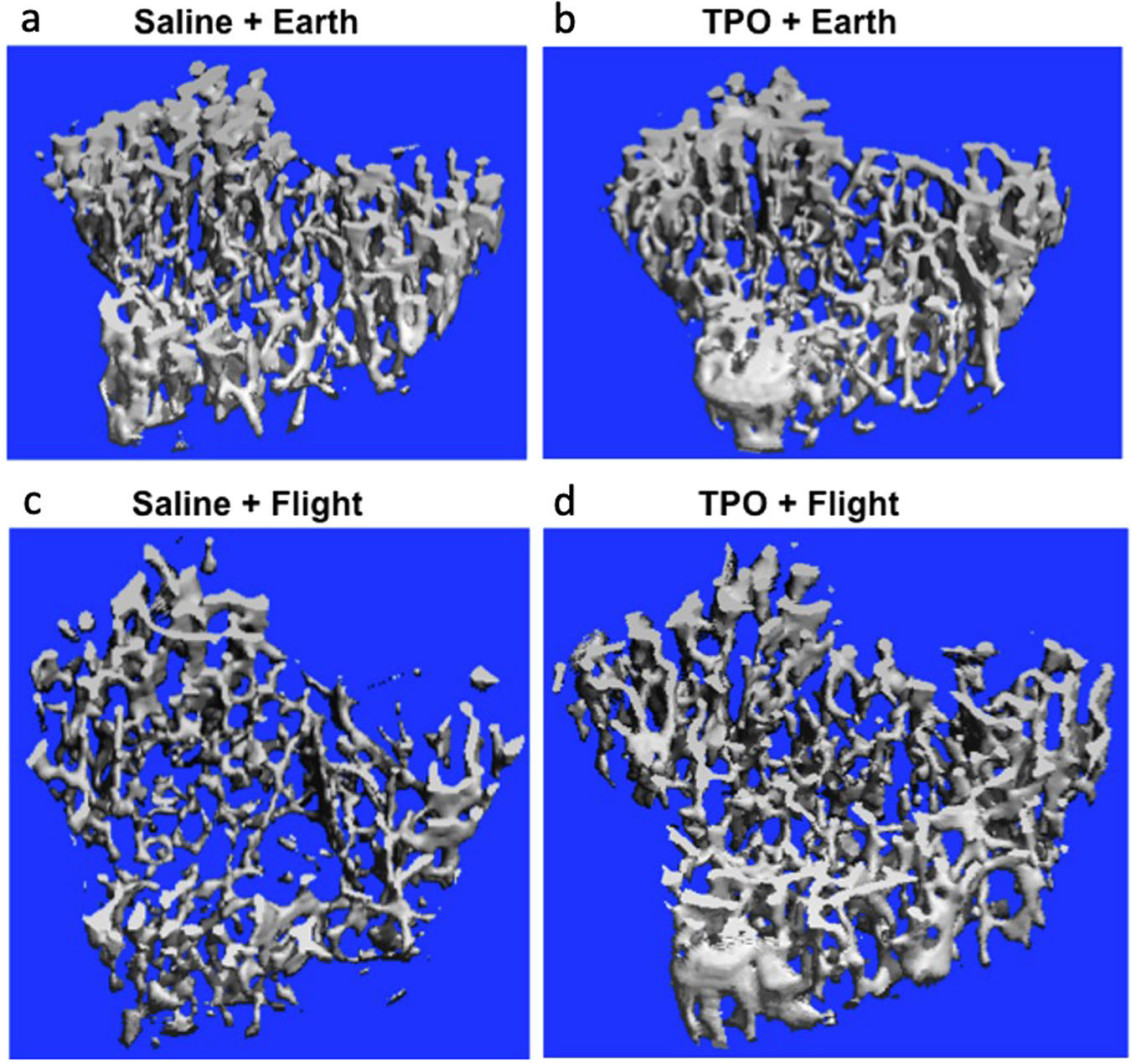
Representative 3D µCT reconstructions of the trabecular compartment of the tibia. **a** Saline + Earth; **b** TPO + Earth; **c** Saline + Flight; and **d** TPO + Flight. Tridimensional microstructural assessment of the trabecular tibia evidencing increments in trabecular number and the connectivity among them in the TPO + Flight group as compared to Saline + Flight group.

#### Cortical tibia (Table 3)

We did not detect any changes in the cortical bone within the tibia due to TPO treatment of femoral fractures on Earth or in flight. Similarly, no differences were observed when comparing saline-treated mice housed on Earth to those in space. However, in the comparison between ground and spaceflight, the exposure to microgravity within the TPO group showed a trend at decreasing total density in flight (−5%, *p* = 0.08). Our two-way ANOVA analyses showed a trend towards a significant TPO × microgravity interaction on T.Ar (*p* = 0.09), B.Ar (*p* = 0.08), and density TV (*p* = 0.08) within the cortical compartment of the tibia.

## Discussion

With the ongoing interest in long-duration spaceflight missions and Mars colonization, unique medical challenges for astronaut crews will occur, including communication delays, the inability to return to Earth early, and the well-known bone loss. A recent study reported significant deficits by 6% in vertebral strength after 6 months of spaceflight, which were not correlated to the 2.2% reduction in BMD. These deficits were not recovered up to 4 years after the mission, leading to a higher risk of vertebral fracture^34^. While the fracture risk after return to the Earth is important, so is the increased risk of fracturing while on spaceflight missions. Although fracture risk appears to be minimal while astronauts are in low Earth orbit, fracture risk is predicted to increase when astronaut are exploring or residing on partial gravity planetary bodies such as the Moon or Mars. Further, predictive models show a high risk of fractures to the hip and wrist in astronauts^15,16^. Therefore, examining fracture healing and fracture treatments in spaceflight, including potential impacts on other skeletal sites, is of critical importance in the preparation of future, longer duration, spacefaring missions.

The bone morphogenetic protein 2 (BMP-2) became a promising therapeutic strategy for repairing bone fractures since its identification in 1988^35^. However, several side effects, including increased risk of developing cancer, have been linked to BMP-2 treatment^30,31^. These side effects have resulted in increased interest in the development of novel and effective, but safer, bone healing therapies. Furthermore, BMP works through mechanical loading pathways^36^, which limits its application to a large variety of diseases and conditions, including space exploration and colonization, as well as disuse related diseases such as paralysis and many others.

TPO is a naturally derived growth factor that induces growth of MKs. TPO and/or MKs are known to regulate skeletal homeostasis and fracture healing^32^. Of importance for this study, it does not appear that TPO’s mechanism of action for fracture healing requires mechanical stimulation. However, TPO as a bone healing therapy has never been tested in space. Considering that pharmacokinetics and pharmacodynamics may be altered in space, we designed this study to understand the systemic effects of TPO on the skeleton in spaceflight. Of note, while there are several pharmacological agents, dietary supplements, and exercises being considered for preventing bone loss in spaceflight^17–21^, it is important to determine whether other agents considered for use in spaceflight for different indications, may impact bone mass parameters. This is an important component of our rationale for investigating the systemic effects of TPO on distant bones after a single, local administration, to induce femoral fracture healing. Therefore, here we used a murine model to analyze the systemic effects of TPO treatment, at the time of surgery, on mice post-femoral fracture, by examining eight other bones in both ground and spaceflight groups. We examined bones from the limbs (humerus and tibia), torso (L4 vertebrae, 10th rib, and 3rd body of the sternum), and skull (calvarium, incisor, and mandible).

Our data evidenced several interesting outcomes with regard to the systemic effects of TPO administration, to a femoral defect injury on distal skeletal sites, in mice housed on Earth and in spaceflight. TPO was the significant main effect at the M.Ar in the mandible; SMI in the sternum; Tb.N and Conn.D in the vertebra; and Tb.Sp and Conn.D at the trabecular humerus. Overall, most of the significant effects of TPO on distal sites were observed in mice housed in space. Specifically, in spaceflight, TPO treatment in the femur increased trabecular number in the vertebral body; in the mandible, TPO decreased marrow area; in the ribs, TPO also decreased marrow area, associated with an increase in bone area fraction; in the sternum, TPO increased trabecular thickness and decreased the spacing between them; in the trabecular humerus, TPO increased the connectivity density and reduced trabecular spacing; in the cortical humerus, TPO decreased the marrow area; and in the trabecular tibia, TPO trended towards an increase in trabecular number and connectivity density. Remarkably, a significant TPO × microgravity interaction was detected in the T.Ar, M.Ar, and B/Ar/T.Ar at the rib; BV/TV and Tb.N at the sternum; and Conn.D at the vertebra. These findings may suggest benefits not only for astronauts, but also for disabled and elderly people, who cannot sustain load to their bones or are restricted in overall mobility.

However, different impacts on distal bones, by TPO delivered to the femur fracture, were observed in mice housed on the Earth. For example, TPO treatment on Earth resulted in an increase in trabecular tissue volume within the humerus; decreased bone volume fraction in the ribs; decreased trabecular number and the bone volume fraction and increased trabecular separation in the sternum; and a decrease in connectivity density in the L4 vertebra.

Furthermore, we identified changes between the TPO-treated mice (Earth versus flight). For the non-weight-bearing bones, we observed higher bone fraction, associated with lower trabecular separation in the calvarium; and lower trabecular separation in the sternum of TPO-treated mice during spaceflight as compared to TPO-treated mice on the Earth. On the other hand, with regard to the trabecular bone within the humerus, we noted lower tissue volume, associated with a higher structure model index in the sternum of TPO-treated mice during spaceflight as compared to TPO-treated mice residing on the Earth. Likewise, TPO-treated mice during spaceflight also exhibited lower total and bone volume, as well as bone volume fraction within the trabecular compartment of the tibia, and a lower density in the cortical compartment of the tibia compared to the TPO-treated group on Earth. We believe this may have happened because TPO, although being a potent osteogenic agent, is not as effective as mechanical loading itself on Earth where mechanosensors are stimulated to form new bone. In our previous studies, we have documented an important and detrimental change in tibial bone geometry, not only due to microgravity exposure, but also due to surgery on the ipsilateral femur^33^, likely owing to reduced weight bearing or systemic surgical effects.

Besides the altered geometry observed in the ipsilateral tibia due to surgery itself, we previously report^33^ how surgery impacted the other bones studied here. Specifically, femoral defect surgery did not result in significant changes in the calvarium, mandible, incisor, vertebra, or humerus, but did impact some parameters in the rib, sternum, and ipsilateral tibia. Interestingly, for the rib and the sternum TPO treatment returns all significantly different parameters back to the unoperated control levels (*p* > 0.1). However for the ipsilateral tibia, TPO treatment does not return trabecular parameters and cortical bone fraction to unoperated control levels (*p* < 0.01), rather values are not different from saline-treated mice (*p* > 0.6).

TPO is considered the primary MK growth factor, which induces human stem cells (HSC) and hematopoietic progenitor cells (HPC) to differentiation into MKs and platelets by activating its receptor, Mpl, and stimulating downstream signaling pathways^37,38^. In this aspect, previous studies have reported that elevated MK numbers lead to improvements in bone mass by stimulating osteoblastic activity^39–46^, and that TPO overexpression in mice is correlated to an increase in MK number and bone mass^47–49^. Furthermore, TPO-Mpl signaling not only plays a role in MK maturation, but also regulates HSC and HPC expansion^37^. Since microgravity has been linked to an improvement in the numbers of HPC and the function of stem cells^50,51^, it is possible that gravity-based changes, and/or TPO-induced changes, could be responsible for altered distal skeletal site bone parameters observed following TPO-treatment during spaceflight than on Earth. In fact, it appears that TPO was able to improve several bone parameters in spaceflown mice as compared to Earth-housed mice, even when administered just once at the time of surgery and only in the femur. Of note, and as previously published by our group, we detected negative effects of spaceflight in all the bones we analyzed with the exception of calvarium and humerus^33,52^. The improvement of several bone parameters in the calvarium is likely owing to the cephalic shift of fluids in space, increasing loading^53,54^. With regard to the humerus, we posit that the increased utilization of the humerus in spaceflight, likely due to mice pushing off with their forearms and grabbing the wire caging, increases the relative loading on the skeleton^33,52^. That said, the overall detrimental effects of unloading on much of the skeleton have been previously reported in several rodent models whereby unloading is used to simulate microgravity (i.e. hindlimb suspension and spinal cord injury), as well in spaceflight missions^9,10,52,55–57^.

Although we have documented that TPO administration at the femoral fracture site alters several bone parameters, which increase bone mass, the mechanisms of action remain unclear, and future studies are required to better address these mechanisms. That said, TPO may act directly on the distal bones by entering the bloodstream, thus accessing the systemic circulation and impacting bone, as has been observed with systemic overexpression of TPO^47–49^. Alternatively, TPO may play an indirect role. Specifically, through its role in bone healing, TPO stimulates cells at the fracture site and the release of cytokines and growth factors which in turn could enter the circulation, leading to systemic effects on distal bones. Further, a combination of both direct and indirect mechanisms may also be impacting the distal skeletal sites. Regardless of the mechanism(s) of action, based on these findings, it does not appear that TPO used for bone-healing indications results in significant negative impacts on distal bones that would be preclude its use as a bone-healing agent in future spaceflight or Earth-based applications.

Spaceflight is a unique environment that poses many barriers to conducting research. Limitations such as access to launch vehicles, the exuberant cost, and keeping live specimens in an orbital laboratory are just some of the many challenges faced. The use of rodent protocols in space minimized many of these challenges. Their small size means less weight per launch, providing the duel benefit of lower cost and a larger sample number. Thus, this spaceflight study sought to examine the systemic effects at distal bone sites following treatment of fractures with a therapy, delivered locally to the fracture site. Furthermore, here we cohoused male mice following our specialized acclimation protocol^58–60^. Notably, prior to our mission, NASA had not cohoused male mice in spaceflight due to their aggressive behavior compared to female mice. Thus, our data also contributes to better understanding the impacts of surgery, spaceflight, and TPO treatment on the male skeleton. Of importance, the spaceflight environment was ideal and necessary to meet the purposes of this study; however, it limited our group sample sizes, as well as the significant differences in our comparisons.

It is critical to highlight that we have used the Benjamini–Hochberg false discovery rate (FDR) in order to control the type I error rate due to the multiple statistical comparisons conducted in this study. We observed that all variables which were reported as being significantly different in Tables 1–3, remained statistically significant after the adjustment of its *p*-value, in which all *p*-values remained lower than their respective FDR (Supplementary Table 1). Therefore, we opted to use the *p*-value reported in Tables 1–3 throughout this manuscript.

In this study, we analyzed several bones in order to make the most out of our spaceflight specimens. Here, we assessed long bones and vertebra, which are widely studied in spaceflight investigations, and also examined lesser studied bones such as the calvarium, mandible, incisor, sternum, and rib. In our previous study, we documented no significant changes at the calvarium when 9 week-old male mice were subjected to spaceflight for ~30 days^52^. Similar findings were also reported by Macaulay et al.^61^ in 20-week-old male mice also exposed to spaceflight for 30 days. However, studies by Zhang et al.^53^ reported increased calvarial bone volume with a trending increase in skull thickness in 23-week-old female mice flown in space for 15 days. In all three of these studies the animals did not undergo surgery. Here, our male spaceflight mice underwent bone fracture surgery and exhibited higher BV/TV compared to Earth mice, irrespective of TPO treatment. This may suggest that female mice and male mice that undergo surgery (or possibly other interventions) exhibit enhanced cephalic fluid shifts in microgravity compared to unoperated male mice. This change highlights the important value of this work at examining lesser studied, yet critically important bones. Especially because previous studies have documented visual impairment, as well as ocular and brain structural changes in astronauts, which are likely caused by the cephalic shift of fluids during spaceflight^62,63^. Furthermore, we encourage further studies to expand upon our investigations in order to better elucidate the effects of spaceflight when combined with additional interventions.

In conclusion, longer spaceflight missions will require optimal treatments for the crew members, in which osteoporotic agents and regenerative therapies to repair bone fractures will be important. Here, we identified that local treatment of femur fractures with TPO improved bone quantity in some parameters at distal skeletal sites in mice subjected to a one-month spaceflight, without exhibiting any grossly observed side-effects. Thus, although more work is required to fully characterize its potential, it appears that TPO may not only enhance bone healing^32^, but may also improve additional bone properties in spaceflight.

## Methods

### Animals and study design

**Fig. 3:**
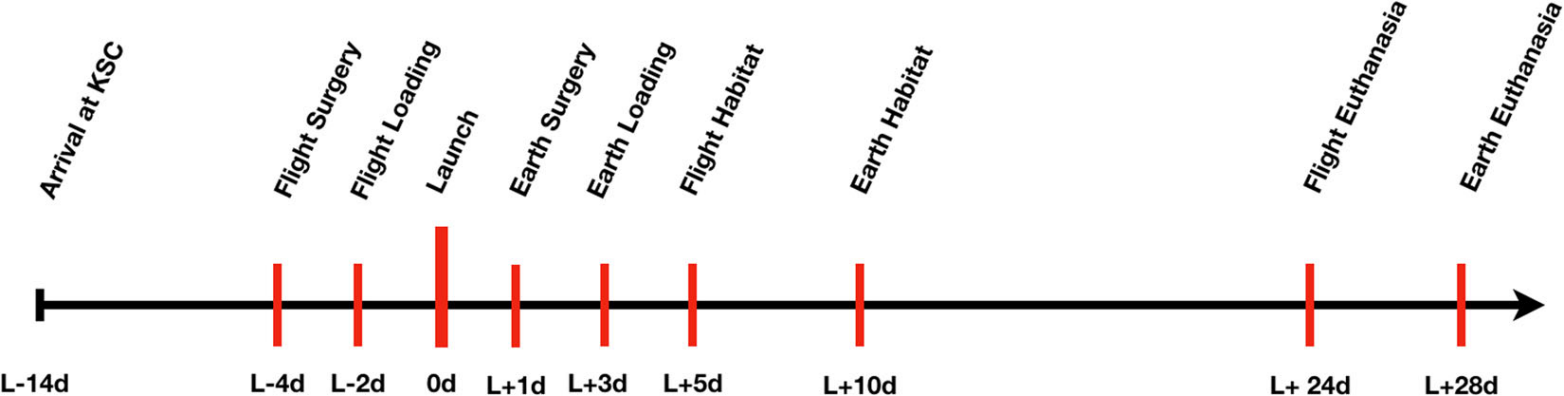
Study timeline. Mice arrived at the Kennedy Space Center (KSC) 14 days prior to launch (L−14d) and were allowed to acclimate to the spaceflight hardware. The Flight groups had their surgeries performed 4 days before launch (L−4d) and 2 days prior to launch they were loaded into spaceflight hardware (Transporters) and loaded onto the SpaceX Dragon Capsule (L−2d). One day after launch asynchronous Earth groups were submitted to identical surgeries (L + 1d). To match the spaceflight conditions, two days after Earth group surgeries, mice were loaded into spaceflight Transporter hardware (L + 3d). Five days after launch, spaceflight mice were transferred by astronauts into the Habitat spaceflight cages aboard the International Space Station, where the mice remained for the study duration (L + 5d). To keep the 5-day asynchronous timing, 10 days after launch, Earth-based mice were transferred into Habitat spaceflight cages at KSC. Four weeks post-surgery euthanasia of mice began over a 5-day period, due to the significant time it takes astronauts to complete these tasks (L + 24d for the Flight groups and L + 28d for the Earth groups).

C57BL/6J male mice were acquired from Jackson laboratories (Bar Harbor, ME) at 7 weeks of age. Housing parameters and acclimation process are described in detail in previous publications^33,52,58–60^. Figure 3 provides a timeline of main experimental details. Briefly, upon arrival at Kennedy Space Center (KSC) the mice were placed in N40 cages with raised wire floors (Ancare, Bellmore, NY). Provisions were provided with the intent to acclimate mice to spaceflight hardware and included modified lixit water bottles and NASA nutrient-upgraded Rodent Food Bar (NuRFB). Mice were weighed twice each week to ensure that they were acclimating to both the NuRFB and water lixit systems used for flight. Mice were ear-punched for identification and maintained in a 12-h night/day cycle in ambient temperatures between 24 and 25 °C. All experimental protocols were approved and performed in accordance with the NIH Guide for the Care and Use of Laboratory Animals (NASA Animal Care and Use Committees, #FLT-15-101/NAS-15-105). This research complied with the Animal Welfare Act and implementing. Animal Welfare Regulations, the Public Health Service Policy on Humane Care and Use of Laboratory Animals and adhered to the principles noted in The Guide for the Care and Use of Laboratory Animals^64^.

### Groups

This data is a part of a larger study examining fracture healing in space aboard the ISS as part of NASA’s Rodent Research 4 (RR4) mission. Here we present data from a subset of the larger parent study. Specifically, data are from mice house on the “Earth” or in spaceflight (“Flight”) and mice treated with either saline as a vehicle control (“Saline”) or Thrombopoietin (“TPO”). Specimens designated as Earth studies were completed 5 days after Flight studies to allow for replication of controlled conditions on the ISS (cage temperature, food and water changes, etc.). After euthanasia, all sample processing was done simultaneously to increase consistent treatment of all specimens. Initially 10 mice were designated per group with each group reserving five specimens for bone studies and five for multiomic analyses. The forelimb group had a larger sample size (*n* = 10) due to both forelimbs remaining intact (e.g. surgery was on the right hindlimb femur). Unfortunately, some groups contained mice that were euthanized early based on NASA veterinarian recommendations. Specifically, in the TPO + Earth group, five mice were euthanized early due to observed aggression and visible wounds on video. There were a few groups in which the sample size was <*n* = 5. In those instances, it was a result of specimen damage or unavailability of 3D microstructure assessment. All specific sample size values are listed in Tables 1–3.

### Surgery

N40 cages contained 15 mice/cage upon arrival to KSC. Cages were randomized into the following four groups: Saline-treated mice housed on Earth (Saline + Earth), TPO-treated mice housed on Earth (TPO + Earth), Saline-treated mice housed on the ISS (Saline + Flight), and TPO-treated mice housed on the ISS (TPO + Flight). A complete surgical procedure has been previously detailed^33,52,58–60^. A condensed outline of the surgical procedure is as follows. Mice were briefly anesthetized with Ketamine-Xyalazine (125–20 mg/kg), once at a surgical plane of anesthesia, the right leg was shaved and then made sterile by scrubbing with betadine and ethanol (three alternating scrubs). The initial incision was lateral and 1 cm in length over the right femoral midshaft. The knee was then flexed, the femoral condyles located, and a 27-gauge intramedullary pin inserted manually between them and threaded retrograde into the intramedullary canal. Next, the pin was removed partially and a 2 mm defect was created over the midshaft of the diaphysis of the femur using a sterile Dremal rotary tool (Dremal Inc., Racine, WI). The defect size was maintained by the insertion of a synthetic graft composed of poly (propylene fumarate)/tricalcium phosphate^65^. The needle was then threaded through the synthetic graft and bored into the greater trochanter via a twisting motion. The exposed needle end was bent backward on itself and pulled distally for stabilization of the femur and defect. The opposite end of the needle was cut as close as possible to the knee. Then collagen sponges (RCM6 Resorbable Collagen Membrane, ACE, Brockton, MA) soaked in saline or 5 μg of TPO for 15 min, were wrapped around the synthetic graft and overlaying native bone and sutured into place (3–0 vicryl suture, Ethicon, Sommerville, NJ). The surrounding muscle was sutured closed and 7 mm wound clips (Braintree Scientific, Braintree, MA) were employed to close the skin. Post-operative monitoring included observation until the mice recovered from anesthesia, administration of analgesic (0.05 mg/kg of buprenorphine), followed by placement of recovered mice into their original cages. Resting boards (K3392 Rest Stops, Bio-Serve, Flemington, NJ) were added into the cages for the first 2 days following surgery. Two days prior to launch the 10 healthiest mice/group (determined by NASA veterinarians in collaboration with MAK and PJC) were transferred into spaceflight hardware (NASA Rodent Transporters which housed the mice while they were on a SpaceX Commercial Dragon and NASA Rodent Habitats which housed mice while they were on the ISS)^66^. Transporter and Habitat footprints are both 59.7 in.^2^, while the N40 cage footprint is 199.5 in.^2^. NASA hardware incorporates wire mesh on all surfaces to make the entire interior accessible to mice and it is referred to as the habitable surface area. The habitable surface area of the Transporter and Habitat are 715 and 882 in.^2^, respectively. The Transporter can house 10 mice on each side (total of 20), while each side of the Habitat can house five mice (total of 10). SpaceX Commercial Dragon can accommodate 2 Transporters, housing up to a total of 20 mice/Transporter (40 total). The ISS can currently accommodate four Habitats, housing up to a total of 10 mice/Habitat (40 total).

### Sample collection

At launch mice were ~9 weeks old, and mice were ~13 weeks old at euthanasia. Astronauts were limited to euthanizing and dissecting 8 mice/day for the parent study, all mice were euthanized between 24 and 28 days post-launch. Euthanasia occurred by injection of ketamine-xylazine (150–45 mg/kg) accompanied by a closed chest cardiac puncture with blood withdrawal and cervical dislocation. Five mice in each group had their right hindlimb removed up to the hip, and the hindlimbs were placed in 10% in neutral buffer formation (NBF) until transfer to 4 °C within ~4–6 h. The samples were kept at these conditions ~2 weeks after euthanasia (until their return to Indiana University School of Medicine or IUSM). The remainder of these carcasses, and the entire carcass from the other 5 mice/group (after cervical dislocation), were wrapped in aluminum foil, frozen, and stored at −80 °C or below on the ISS until their return to the US Army (Fort Detrick, MD) approximately 2 weeks later. Ground mice/carcasses were similarly euthanized/processed 5 days later at KSC and were transferred to ISUM or the US Army at their respective temperatures.

The carcasses were further processed at the US Army as follows. Carcasses were placed on ice blankets for 15 min to partially thaw, tissue dissection for each carcass was performed, and the bones (minus the forelimb) were snap frozen immediately (~45 min out of −80 °C). The removed forelimbs were placed in 10% NBF for 72 h, washed with ice cold PBS and transferred to ice cold 70% ethanol. Forelimb samples were kept at 4 °C during the transfer from the US Army to IUSM and were maintained at that temperature until μCT scanning commenced. The frozen bone specimens were also shipped from the US Army to IUSM on dry ice and were then stored at −80 °C until they were removed for specific dissection as follows. Bone samples were briefly thawed, and the surrounding soft tissue was removed. Then a region of interest (ROI), a standardized anatomical location for analysis, was isolated, fixed in 10% NBF for 72 h at 4 °C, washed with ice cold PBS, and stored in 70% ice-cold ethanol at 4 °C until specimens were subject to micro-computed tomography (µCT) as detailed below.

### Micro-computed tomography

Calvariae, mandibles, incisors, ribs, sternums, vertebrae, and humeri were imaged using a desktop SkyScan 1172 μCT imaging system (SkyScan, Kontich, Germany) and image reconstructions of each specimen were obtained via NRecon v.1.7.3. Bone structure parameters were visualized and determined using Skyscan software, Dataviewer, CTAn (Kontich, Belgium). Tibia were imaged using a desktop SCANCO μCT35 imaging system (SCANCO Medical, Brüttisellen, Switzerland). ROI were dependent on specimen type and are outlined in Table 4. In Table 4 we also detail the instrument settings used and the analyzed bone morphometric parameters, which are in accordance with ASBMR nomenclature^67^. Of note, the specific ROIs for each bone were used to calculate the following parameters. A volume of 100 pixel^3^ centered on the parietal eminence was used as the calvarial ROI to measure tissue volume (TV, in mm^3^), bone volume (BV, in mm^3^), bone volume fraction (BV/TV, in %), marrow volume calculated as TV−BV (MV, in mm^3^), average cortical thickness (Ct.Th, in mm), trabecular thickness (Tb.Th, in mm), trabecular separation (Tb.Sp, in mm), and trabecular number (Tb.N, in 1/mm). The mandibular ROI was defined as the cross-section of a single coronal slice through the middle of the posterior root of the first molar. After subtracting the molar from the region of interest, a 2D analysis was performed on the mandible with the incisor and a subsequent subtraction of the incisor led to the following measurements: tissue area (T.Ar, in mm^2^), bone area (B.Ar, in mm^2^), marrow area calculated as T.Ar−B.Ar (M.Ar, in mm^2^), bone area fraction (B.Ar/T.Ar, in %), and lingual cementum–enamel to alveolar bone crest distance (CEJ–ABC, in mm), which was obtained by measuring the distance from the cementum edge on the lingual tooth surface to the alveolar bone apex. A separate 2D analysis was performed on the incisor alone to calculate T.Ar, enamel + dentin area (E + D.Ar, in mm^2^), pulp area (Pu.Ar, in mm^2^), and enamel + dentin area fraction (E + D.Ar/T.Ar, in %). The shrink-wrap function was used for both the mandible and incisor ROIs to insure accurate T.Ar measurements. For trabecular analyses of the vertebrae and sternum, ROIs were obtained from 1 mm tall segments centered in the L4 vertebral and third sternebral bodies, reporting the following variables: TV, BV, BV/TV, specific bone surface (BS/BV), Tb.Th, Tb.Sp, Tb.N, structure model index (SMI), and connectivity density (Conn.D, in1/μm^3^). A 0.5 mm ROI was obtained from the midshaft of the tenth rib and used to calculate T.Ar, B.Ar, M.Ar, B.Ar/T.Ar, and Ct.Th. For trabecular analysis of the humeri, the ROI started at 0.5 mm distal from the proximal growth plate and extended an additional 0.5 mm distally, calculating TV, BV, BV/TV, BS/BV, Tb.Th, Tb.Sp, Tb.N, SMI, and Conn.D. For the cortical analysis of the humeri, the ROI was set at 0.5 mm proximal from the midshaft and extended an additional 0.5 mm proximally, obtaining T.Ar, B.Ar, M.Ar, B.Ar/T.Ar, and Ct.Th. For trabecular analysis of the tibia, the ROI started at 0.25 mm distal of the proximal growth plate and extended an additional 0.5 mm proximally, calculating TV, BV, BV/TV, Tb.Th, Tb.Sp, Tb.N, SMI, and Conn.D. For cortical analysis of the tibia, a 1 mm ROI was obtained from a region that was 0.25 mm proximal from the tibiofibular junction and the following parameters were measured: T.Ar, B.Ar, M.Ar, B.Ar/T.Ar, Ct.Th, and densities based on both the TV and BV, in 1/cm. The recommended manufacturer low and high thresholds were used for all bone analyses and was selected qualitatively by an experienced operator by comparing segmented trabecular bone to original grayscale images, aiming to obtain a physiologically accurate representation. All instrument parameters and bone analyses parameters are in Table 4. It is important to note that while two different machines were used to complete the analyses and μCT imaging, each skeletal site was scanned using a single machine. This makes comparisons possible without the issue of variation between machines (e.g. the SCANCO system was used for all Earth and Flight, TPO- or Saline-treated tibia).

**Table 4:** Micro-computed tomography settings and parameters for bone analyses.

Bone	Instrument	Parameters
Calvarium	SkyScan 1172 μCT, 60 kV, 5.9 μm voxel	*ROI*: Set as a 100 pixel^3^ volume centered at the parietal eminence. *3D morphometrical parameters*: TV, BV, MV, BV/TV, MV, Ct.Th, Tb.Th, Tb.Sp, and Tb.N. Upper threshold: 255; Lower Threshold: 90
Mandible and Incisor	SkyScan 1172 μCT, 60 kV, 5.9 μm voxel	*ROI*: Single coronal slice taken through the middle of the posterior root of the first molar. This value was subtracted from the ROI prior to 2D analysis on the mandible with the incisor. Separate 2D analysis was performed solely on the incisor as well. For both the mandible and incisor ROIs the shrink-wrap function was used to ensure accurate T.Ar measurements. *Mandible variables*: T.Ar, B.Ar, M.Ar, B.Ar/T.Ar, and CEJ–ABC. Incisor variables: T.Ar, E + D.Ar, Pu.Ar, and E + D.Ar/T.Ar. Upper threshold: 255; Lower Threshold: 120
Rib	SkyScan 1172 μCT, 60 kV, 9.8 μm voxel	*ROI***:** 0.5 mm segment at the midshaft of the tenth rib. *Cortical rib variables*: T.Ar, B.Ar, B.Ar/T.Ar, M.Ar, and Ct.Th. Upper threshold: 255; Lower Threshold: 90
Sternum	SkyScan 1172 μCT, 60 kV, 5.9 μm voxel	*ROI*: 1 mm tall segments centered at the third sternebral body. *3D analyses variables*: TV, BV, BV/TV, BS/BV, Tb.Th, Tb.Sp, Tb.N, SMI, and Conn.D. Upper threshold: 255; Lower Threshold: 90
Vertebra	SkyScan 1172 μCT, 60 kV, 5.9 μm voxel	*ROI*: 1 mm tall segments centered at L4 vertebral body. *3D analyses variables***:** TV, BV, BV/TV, BS/BV, Tb.Th, Tb.Sp, Tb.N, SMI, and Conn.D. Upper threshold: 255; Lower Threshold: 110
Trabecular humerus	SkyScan 1172 μCT, 60 kV, 5.9 μm voxel	*ROI***:** 0.5 mm segment distal from proximal growth plate and extended an additional 0.5 mm distally. *3D analyses***:** TV, BV, BV/TV, BS/BV, Tb.Th, Tb.Sp, Tb.N, SMI, and Conn.D. Upper threshold: 255; Lower Threshold: 80
Cortical humerus	SkyScan 1172 μCT, 60 kV, 5.9 μm voxel	*ROI*: 0.5 mm proximal from the midshaft and extended an additional 0.5 mm proximally to avoid the deltoid tuberosity. *2D analyses***:** T.Ar, B.Ar, M.Ar, B.Ar/T.Ar, and Ct.Th. Upper threshold: 255; Lower Threshold: 110
Trabecular tibia	SCANCO μCT35, 55 kV, 12 μm voxel	*ROI***:** Region 0.25 mm distal of the proximal growth plate and extended an additional 0.5 mm proximally. In this instance ROI is also the tissue volume (TV). *Variables***:** TV, BV, BV/TV, Tb.Th, Tb.Sp, Tb.N, SMI, and Conn.D.
Cortical tibia	SCANCO μCT35, 55 kV, 12 μm voxel	*ROI***:** 1 mm segment obtained from region 0.25 mm proximal from the tibiofibular junction. The area contained within the periosteal surface is the cortical TV. *Variables***:** T.Ar, B.Ar, M.Ar, B.Ar/T.Ar, Ct.Th and density. Calculations for B.Ar, M.Ar, and T.Ar are as follows: B.Ar = BV/ height of the analyzed bone segment (1 mm). Marrow area (M.Ar) and tissue area (T.Ar) were calculated by the equation for the area of a cylinder: B.Ar = π*(total radius^2^−marrow radius^2^). By substituting total radius = Ct.Th + marrow radius and solving for marrow radius, we obtained the following equation: marrow radius = ((B.Ar/*π*)−Ct.Th^2^)/(2*Ct.Th). Next, M.Ar was calculated as π*marrow radius^2^, and T.Ar was calculated as B.Ar + M.Ar.

All relevant data concerning instrument settings, ROI, variables and their derivations for each bone analyzed. Variables recorded include: tissue volume (TV, in mm^3^), bone volume (BV, in mm^3^), bone volume fraction (BV/TV, in %), marrow volume calculated as TV−BV (MV, in mm^3^), average cortical thickness (Ct.Th, in mm), trabecular thickness (Tb.Th, in mm), trabecular separation (Tb.Sp, in mm), trabecular number (Tb.N, in 1/mm), tissue area (T.Ar, in mm^2^), bone area (B.Ar, in mm^2^), marrow area calculated as T.Ar−B.Ar (M.Ar, in mm^2^), bone area fraction (B.Ar/T.Ar, in %), lingual cementum–enamel to alveolar bone crest distance (CEJ–ABC, in mm), enamel + dentin area (E + D.Ar, in mm^2^), pulp area (Pu.Ar, in mm^2^), enamel + dentin area fraction (E + D.Ar/T.Ar, in %), specific bone surface (BS/BV), structure model index (SMI), connectivity density (Conn.D, 1/μm^3^, with the exception of tibia—1/mm^3^).

### Statistical analyses

Continuous variables were expressed as the mean and standard deviation (SD). All data were tested for normality using the Kolmogorov–Smirnov test. Two-way ANOVAs followed by Tukey post-hoc analyses were used to detect significant differences for comparisons between (1) groups housed under the same gravitational condition to understand the effects of treatment both on Earth and in flight (i.e. Saline + Earth versus TPO + Earth or Saline + Flight versus TPO + Flight), as well as (2) comparisons between spaceflight and Earth housed mice to assess the influence of gravity on the treatment (i.e. TPO + Earth versus TPO + Flight or Saline + Earth versus Saline + Flight). *p* values less than 0.05 were considered statistically significant. We also discuss trending data (*p* < 0.09) in order to highlight potentially interesting areas for future inquiry. In order to control the type I error rate from multiple comparisons, we have used the Benjamini–Hochberg false discovery rate (FDR, Supplementary Table 1). All statistical analyses were performed with RStudio 1.3 (RStudio, Inc., USA).

### Reporting summary

Further information on research design is available in the Nature Research Reporting Summary linked to this article.

## Supplementary information

Supplementary Table 1

Reporting Summary Checklist

## Data Availability

The data that support the findings of this study are available from the corresponding author upon reasonable request.
