# Supplementary material for: Analysis of the effects of spaceflight and local administration of thrombopoietin to a femoral defect injury on distal skeletal sites
Source: NPJ Microgravity. 2021 Mar 26;7:12. doi: 10.1038/s41526-021-00140-0 (PMC7997973; doi:10.1038/s41526-021-00140-0)
Supplement: Supplementary file 1 — Supplementary Table 1 [file 41526_2021_140_MOESM1_ESM.pdf]

Supplemental Table 1 Summary of two-way ANOVA analysis and adjusted p-value by the Benjamini-Hochberg False Discovery Rate

Bone	Variable	Comparison/Main effect/Interaction	p-value	FDR
Calvarium	BV/TV	TPO+Flight vs Saline+Flight	0.025	0.214
	MV	TPO+Flight vs Saline+Flight	0.024	0.214
	Ct.Th	TPO+Flight vs Saline+Flight	0.049	0.214
	Tb.Th	TPO+Flight vs Saline+Flight	0.031	0.214
	Tb.Sp	TPO+Flight vs Saline+Flight	0.029	0.214
	Tb.N	TPO+Flight vs Saline+Flight	0.044	0.214
	BV/TV	Saline+Earth vs Saline+Flight	0.016	0.067
	MV	Saline+Earth vs Saline+Flight	0.017	0.067
	Ct.Th	Saline+Earth vs Saline+Flight	0.016	0.067
	Tb.Th	Saline+Earth vs Saline+Flight	0.008	0.067
	Tb.Sp	Saline+Earth vs Saline+Flight	0.014	0.067
	Tb.N	Saline+Earth vs Saline+Flight	0.018	0.067
	BV/TV	TPO+Earth vs TPO+Flight	0.071	0.353
	Tb.Sp	TPO+Earth vs TPO+Flight	0.085	0.353
	BV/TV	Microgravity main effect	0.002	0.005
	MV	Microgravity main effect	0.003	0.009
	Ct.Th	Microgravity main effect	0.002	0.006
	Tb.Th	Microgravity main effect	0.001	0.004
	Tb.Sp	Microgravity main effect	0.001	0.005
	Tb.N	Microgravity main effect	0.003	0.010
Mandible	M.Ar	TPO+Flight vs Saline+Flight	0.041	0.197
	M.Ar	TPO main effect	0.032	0.097
Rib	B.Ar/T.Ar	TPO+Earth vs Saline+Earth	0.019	0.222
	T.Ar	TPO+Flight vs Saline+Flight	0.028	0.133
	B.Ar	TPO+Flight vs Saline+Flight	0.032	0.133
	M.Ar	TPO+Flight vs Saline+Flight	0.035	0.133
	T.Ar/B.Ar	TPO+Flight vs Saline+Flight	0.065	0.184
	T.Ar	Saline+Earth vs Saline+Flight	0.085	0.353
	M.Ar	Saline+Earth vs Saline+Flight	0.093	0.353
	B.Ar/T.Ar	Saline+Earth vs Saline+Flight	0.017	0.199
	B.Ar/T.Ar	TPO+Earth vs TPO+Flight	0.064	0.653
	T.Ar	TPO x Microgravity interaction	0.012	0.037
	M.Ar	TPO x Microgravity interaction	0.039	0.117
	B.Ar/T.Ar	TPO x Microgravity interaction	0.005	0.016
Sternum	BV/TV	TPO+Earth vs Saline+Earth	0.070	0.571
	Tb.Sp	TPO+Earth vs Saline+Earth	0.042	0.539
	Tb.N	TPO+Earth vs Saline+Earth	0.022	0.539
	Tb.Th	TPO+Flight vs Saline+Flight	0.038	0.978
	Tb.Sp	TPO+Flight vs Saline+Flight	0.049	0.978
	BV/TV	Saline+Earth vs Saline+Flight	0.045	0.712
	BS/BV	Saline+Earth vs Saline+Flight	0.090	0.767
	Tb.N	Saline+Earth vs Saline+Flight	0.049	0.712
	Tb.Sp	TPO+Earth vs TPO+Flight	0.072	1.000
	TV	TPO main effect	0.085	0.254
	Tb.N	TPO main effect	0.063	0.094
	SMI	TPO main effect	0.045	0.136
	BV/TV	TPO x Microgravity interaction	0.029	0.087
	Tb.N	TPO x Microgravity interaction	0.027	0.081
L4	Conn.D	TPO+Earth vs Saline+Earth	0.024	0.601

	Tb.N	TPO+Flight vs Saline+Flight	0.044	0.601
	Conn.D	Saline+Earth vs Saline+Flight	0.022	0.566
	Tb.Sp	TPO main effect	0.066	0.199
	Tb.N	TPO main effect	0.046	0.139
	Conn.D	TPO main effect	0.032	0.047
	Conn.D	Microgravity main effect	0.066	0.066
	Conn.D	TPO x Microgravity interaction	0.008	0.024
Trabecular humerus	TV	TPO+Earth vs Saline+Earth	0.034	0.813
	BV	TPO+Earth vs Saline+Earth	0.064	0.329
	Tb.Sp	TPO+Flight vs Saline+Flight	0.014	0.343
	Conn.D	TPO+Flight vs Saline+Flight	0.027	0.343
	TV	TPO+Earth vs TPO+Flight	0.013	0.321
	SMI	TPO+Earth vs TPO+Flight	0.044	0.539
	Tb.Sp	TPO main effect	0.006	0.018
	Conn.D	TPO main effect	0.047	0.071
	Conn.D	Microgravity main effect	0.019	0.056
Cortical humerus	M.Ar	TPO+Flight vs Saline+Flight	0.040	0.417
	M.Ar	Saline+Earth vs Saline+Flight	0.018	0.202
	B.Ar/T.Ar	Saline+Earth vs Saline+Flight	0.071	0.354
	M.Ar	Microgravity main effect	0.011	0.034
	M.Ar	TPO x Microgravity interaction	0.063	0.094
Trabecular tibia	BV	Saline+Earth vs Saline+Flight	0.073	0.499
	Tb.N	Saline+Earth vs Saline+Flight	0.054	0.499
	BV	TPO+Earth vs TPO+Flight	0.029	0.229
	BV/TV	TPO+Earth vs TPO+Flight	0.031	0.229
	Tb.Th	TPO+Earth vs TPO+Flight	0.013	0.229
	TV	Microgravity main effect	0.084	0.251
	Tb.Sp	Microgravity main effect	0.090	0.269
	Tb.N	Microgravity main effect	0.083	0.206
	Tb.Th	TPO main effect	0.065	0.098
	Tb.Th	TPO x Microgravity interaction	0.063	0.098
Cortical tibia	Density TV	TPO+Flight vs Saline+Flight	0.082	0.762
	T.Ar	TPO x Microgravity interaction	0.086	0.259
	B.Ar	TPO x Microgravity interaction	0.080	0.240
	Density TV	TPO x Microgravity interaction	0.076	0.228
