# Supplementary material for: Analysis of the effects of spaceflight and local administration of thrombopoietin to a femoral defect injury on distal skeletal sites
Source: NPJ Microgravity. 2021 Mar 26;7:12. doi: 10.1038/s41526-021-00140-0 (PMC7997973; doi:10.1038/s41526-021-00140-0)
Supplement: Supplementary file 2 — Reporting Summary Checklist [file 41526_2021_140_MOESM2_ESM.pdf]

nature research

Corresponding author(s): Melissa Kacena

Reporting Summary

Nature Research wishes to improve the reproducibility of the work that we publish. This form provides structure for consistency and transparency in reporting. For further information on Nature Research policies, see Authors & Referees and the Editorial Policy Checklist.

Statistical parameters

When statistical analyses are reported, confirm that the following items are present in the relevant location (e.g. figure legend, table legend, main text, or Methods section).

n/a	Confirmed	
☐	☒	The exact sample size (*n*) for each experimental group/condition, given as a discrete number and unit of measurement
☐	☒	An indication of whether measurements were taken from distinct samples or whether the same sample was measured repeatedly
☐	☒	The statistical test(s) used AND whether they are one- or two-sided<br>*Only common tests should be described solely by name; describe more complex techniques in the Methods section.*
☐	☒	A description of all covariates tested
☐	☒	A description of any assumptions or corrections, such as tests of normality and adjustment for multiple comparisons
☐	☒	A full description of the statistics including central tendency (e.g. means) or other basic estimates (e.g. regression coefficient) AND variation (e.g. standard deviation) or associated estimates of uncertainty (e.g. confidence intervals)
☒	☐	For null hypothesis testing, the test statistic (e.g. *F*, *t*, *r*) with confidence intervals, effect sizes, degrees of freedom and *P* value noted<br>*Give P values as exact values whenever suitable.*
☒	☐	For Bayesian analysis, information on the choice of priors and Markov chain Monte Carlo settings
☒	☐	For hierarchical and complex designs, identification of the appropriate level for tests and full reporting of outcomes
☒	☐	Estimates of effect sizes (e.g. Cohen's *d*, Pearson's *r*), indicating how they were calculated
☒	☐	Clearly defined error bars<br>*State explicitly what error bars represent (e.g. SD, SE, CI)*

Our web collection on statistics for biologists may be useful.

Software and code

Policy information about availability of computer code

Data collection	Calvaria, mandible, incisor, ribs, sternum, vertebrae and humerus images for each specimen were reconstructed with NRecon v.1.7.3 and CTAn v1.17.7.2 (Skyscan Software, Kontich, Belgium) . Femur and tibia were imaged and reconstructed using SCANCO Medical microCT systems software suite (SCANCO Medical, Brüttisellen, Switzerland).
Data analysis	All statistical analyses were performed with RStudio 1.0.153 (RStudio, Inc., USA).

For manuscripts utilizing custom algorithms or software that are central to the research but not yet described in published literature, software must be made available to editors/reviewers upon request. We strongly encourage code deposition in a community repository (e.g. GitHub). See the Nature Research guidelines for submitting code & software for further information.

Data

Policy information about availability of data

All manuscripts must include a data availability statement. This statement should provide the following information, where applicable:

- Accession codes, unique identifiers, or web links for publicly available datasets
- A list of figures that have associated raw data
- A description of any restrictions on data availability

The data that support the findings of this study are available from the corresponding author upon reasonable request.

April 2018

Field-specific reporting

Please select the best fit for your research. If you are not sure, read the appropriate sections before making your selection.

☒ Life sciences ☐ Behavioural & social sciences ☐ Ecological, evolutionary & environmental sciences

For a reference copy of the document with all sections, see nature.com/authors/policies/ReportingSummary-flat.pdf

Life sciences study design

All studies must disclose on these points even when the disclosure is negative.

Sample size	As this was a spaceflight study, sample size was limited due to spaceflight hardware constraints. We began with 5 or 10 specimens/group but as the experiment proceeded some specimens were lost as detailed in the manuscript.
Data exclusions	Data were only excluded if specimens were damaged or could not be processed as described in the manuscript.
Replication	The experiment was not repeated as this was a spaceflight, but there were 10 mice in each group as detailed in the manuscript. All attempts were made to ensure data are reproducible and proper controls were performed with equipment etc.
Randomization	Mice were randomized into groups by cages.
Blinding	Investigators were blinded to groups during sample collection and data processing. Groups were unblinded for statistical analysis to identify which groups were being compared to one another.

Reporting for specific materials, systems and methods

Materials & experimental systems

n/a	Involved in the study
☒	☐ Unique biological materials
☒	☐ Antibodies
☒	☐ Eukaryotic cell lines
☒	☐ Palaeontology
☐	☒ Animals and other organisms
☒	☐ Human research participants

Methods

n/a	Involved in the study
☒	☐ ChIP-seq
☒	☐ Flow cytometry
☒	☐ MRI-based neuroimaging

Animals and other organisms

Policy information about studies involving animals; ARRIVE guidelines recommended for reporting animal research

Laboratory animals	C57BL/6J male mice were acquired from Jackson laboratories (Bar Harbor, ME) at 7 weeks of age.
Wild animals	This study did not involve wild animals.
Field-collected samples	This study did not involve collection of field samples.
